# Serial Measurements of Refractive Index, Glucose and Protein to Assess Gastric Liquid Nutrient Transport—A Proof-of-Principal Study

**DOI:** 10.3389/fnut.2021.742656

**Published:** 2022-02-03

**Authors:** Matthias Wittstock, Matthias Kästner, Stephan Kolbaske, Tina Sellmann, Katrin Porath, Robert Patejdl

**Affiliations:** ^1^Department of Neurology, University Medical Center Rostock, Rostock, Germany; ^2^Oscar Langendorff Institute of Physiology, University Medical Center Rostock, Rostock, Germany

**Keywords:** gastric emptying, gastrointestinal motility, refractometry, enteral feeding, stroke, feeding intolerance, critical illness

## Abstract

Delayed gastric emptying contributes to complications as aspiration or malnutrition. Among patients suffering from acute neurological diseases, motility disorders are prevalent but poorly understood. Thus, methods to measure gastric emptying are required to allow for appropriate adaptions of individual enteral nutrition algorithms. For enterally fed patients repetitive concentration measurements of gastric content have been proposed to assess gastric emptying. This approach can be used to calculate the gastric residual volume (GRV) and transport of nutrition formula (NF), but it has not yet been implemented in clinical routine. The aim of this study was to investigate whether refractometry or other likewise straightforward analytical approaches produce the best results under *in vitro* conditions mimicking the gastric milieu. We measured NF in different known concentrations, either diluted in water or in simulated gastric fluid (SGF), with each of the following methods: refractometer, handheld glucose meter, and Bradford protein assay. Then, in enterally fed patients suffering from acute neurological disease, we calculated GRVs and nutrition transport and tested possible associations with clinical parameters. In water dilution experiments, NF concentrations could be assessed with the readout parameters of all three methods. Refractometry yielded the most precise results over the broadest range of concentrations and was biased least by the presence of SGF (detection range for Fresubin original fibre, given as volume concentration/normalized error of regression slope after incubation with water or SGF: 0–100 vs. 0–100%/0.5 vs. 3.9%; glucose-measurement: 5–100 vs. 25–100%/7.9 vs. 6.1%; Bradford-assay: 0–100 vs. 0–100%/7.8 vs. 15.7%). Out of 28 enterally fed patients, we calculated significant slower nutrition transport in patients with higher blood glucose (Rho −0.391; *p* = 0.039) and in patients who received high-dose sufentanil (Rho −0.514; *p* = 0.005). Also, the calculated nutrition transport could distinguish patients with and without feeding intolerance (Median 6 vs. 17 ml/h; Mann-Whitney test: *p* = 0.002). The results of our study prove that serial refractometry is a suitable and cost-effective method to assess gastric emptying and to enhance research on gastrointestinal complications of stroke.

## Introduction

Gastric motility disorders give rise to clinical complications as feeding intolerance, abdominal pain, vomiting, and altered pharmacokinetics for orally administered drugs (1–5). In addition, gastroparesis may increase the risk of aspiration in ventilated patients and the risk of hypoglycemia in diabetic subjects (6, 7). In patients suffering from chronic gastroparesis, changes in functional parameters occur rather slowly within a time range of several months up to years, whereas changes in gastric emptying caused by critical illness or acute neurologic disease may occur within hours to days (8–10).

Although a number of methods for evaluating gastric function have been developed over the last decades, they are not routinely used in the setting of critical care, most likely to their perceived costs, complexity, or time efforts (11–13).

The population of critically ill patients is prone to suffer cohort-specific complications from gastroparesis, since nutrition in sedated patients is not regulated by physiological behavioral feedback mechanisms. Instead medical staff controls nutrition intake according to the local feeding regime (14, 15). This bears the risk of exceeding the transport capacity of the stomach. The estimation of gastric residual volumes (GRV) by mere aspiration of contents (“aspired GRV”) has been shown to be an insufficient parameter with poor correlation to feeding complication and gastric transport (16–19).

Accurate information on the state of gastric function is fundamental to optimizing alimentation and reducing complications. Since gastric motility in critical illness underlies dynamic changes, it is desirable that such information may become easily available and be measured repeatedly without restrictions at the point of care, comparable to blood glucose testing, or blood gas analysis. These requirements are, however, not met by scintigraphy, the current “gold standard” methods for assessing gastric emptying (11, 13). Although it can rather easily be applied if a mobile gamma-camera is available, the handling of the used isotopes and principal reservations to radioactivity among patients and medical staff may limit its use. In addition, scintigraphy provides reliable information on gastric emptying, but not of GRV. In contrast, imaging techniques as sonography or magnetic resonance imaging allow only observations of GRV and not of nutrition transport rates. Sonography also requires a high degree of training while the reliability in obese patients is limited (11, 13).

Another method to measure gastric emptying is based upon the kinetics at which stable isotopes (C^13^) appear in expired air after ingestion of a meal containing isotope labeled fatty acids. The method has the advantage of being non-invasive, but is, as discussed in Kar et al. (13), prone to give rather inaccurate results in critically ill patients due to its dependence on physiological resorption in the duodenum and because of the measurement errors which occur when gastric emptying is slowed markedly and only very low amounts of isotope can be recovered for analysis. Nevertheless, Chapman et al. found a good correlation of results when assessing liquid meal emptying of critically ill patients with both scintigraphy and breath test (20).

Serial measurements of gastric content following strictly defined protocols including applications of indicator substances and water have been shown to be accurate and practical in critically ill patients. A special advantage is the use of the nasogastric tube and nutrition formula (NF) that patients are supplied with anyway for routine care, so additional interventions are not necessary at all (21).

In their review from 2009, Moreira and McQuiggan concluded that serial refractometry indeed was the best suited method for measuring gastric emptying in critically ill patients (11). This recommendation was based largely upon the fact that refractometry fulfills the aforementioned requirements regarding practicability and repeatability.

Refractometry is based upon changes in optical density of fluids that occur with increasing concentrations of non-water molecules. The term “optical density” or “refractive index” describes the decrease in the speed at which light passes the respective medium compared to the speed of light in vacuum. The degree to which solvents increase the optical density of a solution is substance-specific and concentration-dependent. It thus follows that samples of mixed solutions in which certain components show different concentrations will have different optical densities and that, vice versa, concentrations of substances can be estimated from differences in the optical density of that fluid. This fact has been used for many decades in the production of alcoholic beverages or in chemical and clinical analysis of urine or blood serum samples. Under certain conditions, the optical density is indicative of absolute concentrations. For instance, the “Brix Value” (BV) can be read directly from handheld refractometers: 1° Brix Value indicates a concentration of 1 g sucrose per 100 g of sample solution, i.e., of an ideal pure water/sucrose mixture. In less well-defined solutions, the BV will not represent sucrose concentration, but its changes will still correlate with changes in the overall concentration of solvent in the tested fluid (22).

The practicability of refractometry has been demonstrated in a series of studies from the group of Chang (16, 21, 23–26): First of all, this group could demonstrate that it is possible to accurately measure volume concentrations of various single and mixed ingredients of common solutions used for enteral nutrition diluted with water or fasting gastric juice under *in vitro* conditions using simple handheld refractometers. They also systematically varied the pH and the temperature of the mixture (21). In subsequent studies, the same group applied refractometry to patients receiving bolus or continuous tube feeding. Thereby, they introduced mathematical formulas for calculating GRV and NF-concentration in gastric juice and, derived from both, the absolute amount of NF present in the stomach. The latter is of special importance, since repeated measurements of the absolute formula content remaining in the stomach allow direct estimates of gastric nutrient- and thus energy transport (16, 23, 24). Following works essentially reproduced these findings without adding significant new aspects (25, 26).

The focus of all these studies was to demonstrate the utility of refractometry in monitoring enteral nutrition in a mixed and only scarcely characterized group of patients. To our best knowledge, neither the underlying diseases of the patients nor the presence or absence of classical risk factors for altered gastric motility were analyzed in any of these works. We are not aware of any published works in which the results of Chang et al. have been reproduced by other groups or in which this simple and easily applicable approach has been used to study the risk of gastric dysmotility in specific patient populations or to evaluate the impact of distinct risk factors and preventive or even therapeutic strategies.

With the present study, we intend to continue and refine the use of refractometry in the field of clinical nutrition of critically ill patients, both for monitoring individual feeding regimen and as an easily applicable tool for assessing gastric dysmotility in clinical studies.

Two limitations of refractometry which might by now have impeded its widespread implementation have to be discussed:

First, although suitable handheld refractometers are cheap and robust, they are not certified for medical diagnostic procedures. This point may hamper the acceptance of the method by medical staff and be a relevant reason why refractometry is still—despite its cost effectiveness, simplicity, and the convincing data—not widely used in clinical practice.

The second limitation is a theoretical: The mathematical equations used to calculate transport rates using refractometry rely on the assumption that gastrointestinal secretions in the stomach have a refractive index very close to zero and thus do not contribute relevantly to the mean refractive index of the water-NF-mixture (21). This, however, is not fully correct: Hydrochloric acid (HCl), mucins, and pepsin (but not glucose) occur in fasting gastric fluid (27, 28) and may increase the refractive index (BV) to values higher than zero, though still significantly lower than that of the NF that is used as tracer substance. Higher BV and higher amounts of secrete in the stomach would lead to an overestimation of the NF contained in the sample. The Supplementary Figure 1 depicts the amount of overestimation as a function of the two parameters for a scenario which equals the described procedure of this study, i.e., of adding 50 ml of NF as the basal indicator for further dilutions.

Considering that gastric secretion rates of 180 ml/h are not uncommon and that Chang et al. report the BV of gastric juice to be about 2.0, it is evident that relevant bias by this factor cannot be ruled out (21, 29). Therefore, it seemed reasonable to us to evaluate the use of specific substances as indicators instead of the BV. It seemed ideal to us to choose ingredients of the NF which are not secreted by the stomach in relevant numbers and which are still easy to measure. Glucose and total protein seemed to meet these prerequisites in our eyes, since they are not secreted in relevant amounts by the gastric mucosa (30, 31).

Furthermore, the mathematical formulas introduced by Chang (21) for calculating NF content rely on a second assumption: It is assumed that the refractive index of a given volume of formula—fluid—mixture is constant over time under the conditions present in the human stomach. It has, however, not been tested whether hydrolysis under conditions of low pH and proteolysis by pepsin may alter the BV of the mixture. To our knowledge, the impact of possible deviations from these assumptions on the accuracy of measurements has not yet been thoroughly discussed or investigated in a study.

In the present study, we performed *in vitro* experiments to assess the practicability of measuring protein and glucose concentrations with defined dilutions of NF in water or in simulated gastric fluid (SGF) as possible alternatives to refractive index estimations. Especially in the case of glucose, the theoretical advantage of a zero-concentration in gastric fluid under fasting conditions combines with the possibility of using already ubiquitously existing, approved, and quality-checked devices for its measurement. In these experiments, it turned out that refractometry was confirmed as the optimum method for assessing gastric volumes and transport. In a cohort of patients suffering from acute neurological diseases, we could thereafter corroborate the findings of Chang et al. reported in other settings (21, 26) and prove its significance to identify patients with impaired gastric emptying.

## Materials and Methods

### Formula Dilution Experiments: Refractometry

Water and formula solution for enteral nutrition from different suppliers were thoroughly mixed to produce defined volume concentrations. The NF products tested were Fresubin Original Fiber and Diben Drink (Fresenius Kabi Germany, Bad Homburg) as well as Osmolite plus (Abbott Nutrition, Dublin, Ireland). Subsequently, three single droplets of mixture were then put on the prism of the refractometer (PCE-032, PCE Germany, Meschede) and the lid was closed carefully to avoid formation of air bubbles.

The BV was read and documented immediately for the first and all subsequent samples. The procedure was repeated three times for each type of formula. The procedure was then performed with SGF instead of water. Fresh SGF was produced according to published protocols (32). Briefly, 2 g NaCl were dissolved with 3.2 g pepsin (both Sigma-Aldrich, Germany) and 7 ml of 37% HCl (Carl Roth, Germany) in double distilled water to give a total volume of 1,000 ml at an adjusted pH of 1.2 (Schott-Geräte GmbH, Germany).

### Formula Dilution Experiments: Glucose Measurements

Aliquots of formula (Fresubin original fibre) and either water or SGF were prepared in the same manner as for refractometry. After mixing formula and water, a single droplet of the mixture was brought in contact with a glucose test strip which was positioned in an activated handheld glucose meter (Contour next, Bayer Vital GmbH, Germany).

### Formula Dilution Experiments: Protein Measurements

Protein concentration was measured using a Bradford-assay. Samples of casein in water and SGF were prepared and used for validating the assay prior to experiments with NF. Then, NF (Fresubin Original Fiber) mixed with water or SGF was produced as already described and 100 μl of these aliquots were then mixed with 900 μl of Bradford reagent (Sigma-Aldrich, Germany). After 5 min of incubation, extinction was measured at 595 nm using a photometer (BioPhotometer 6131, Eppendorf, Germany).

### Measurements of Brix Values of Patient Samples

**Figure 1:**
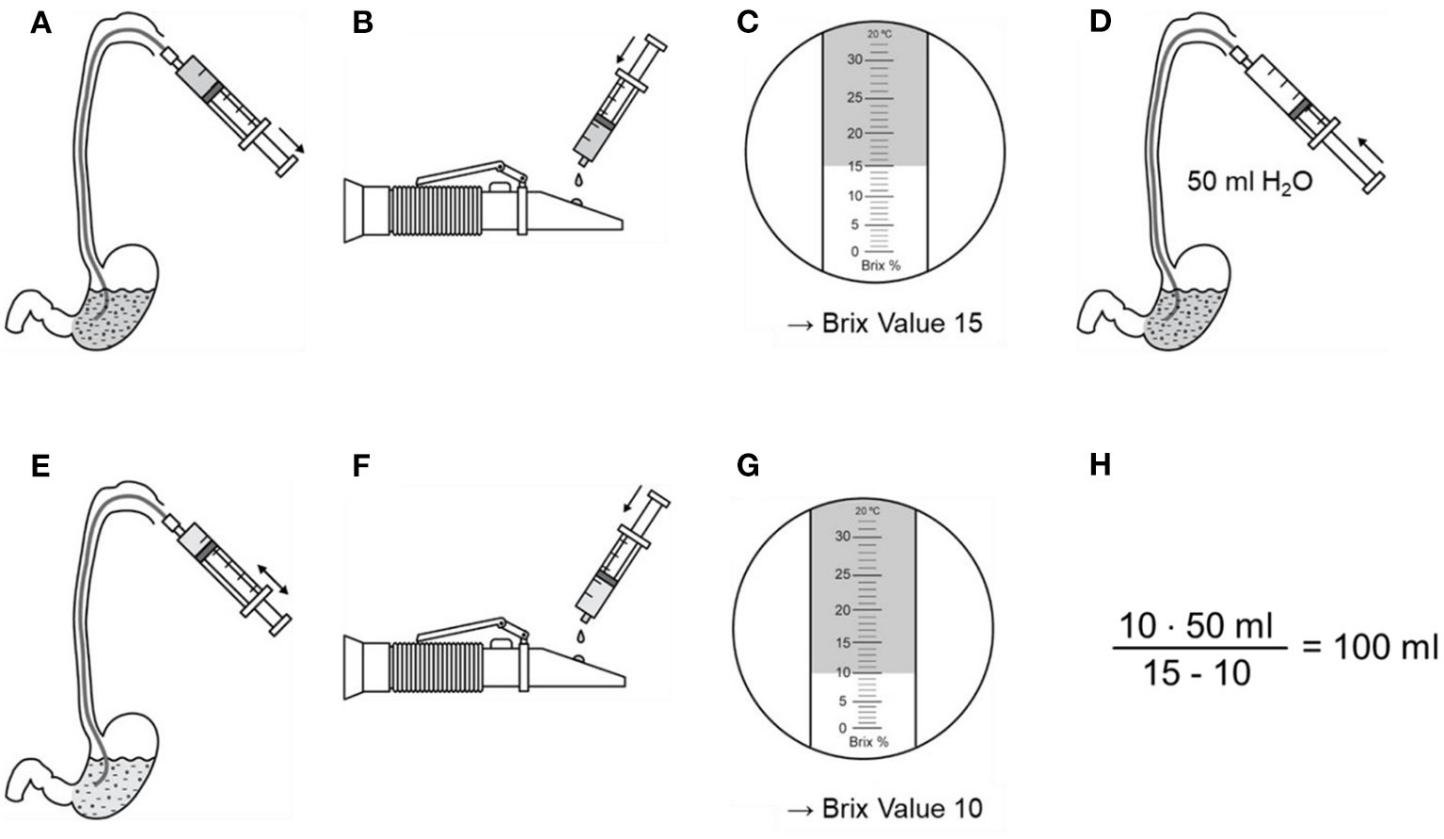
Example for measurement and calculation of gastric residual volume. **(A)** Two milliliters of gastric content are aspirated via a nasogastric tube and **(B)** dripped at the refractometer. **(C)** The Brix Value is read. **(D)** Gastric content is diluted with 50 ml water and mixed well. **(E)** Two milliliters are aspirated and **(F)** dripped at the refractometer again. **(G)** Gastric residual volume is now calculated using the Brix Value before and after dilution, as well as the volume of water.

Patients were considered eligible for inclusion if they had been supplied with a nasogastric feeding tube or percutaneous endoscopic gastrostomy for medical reasons, e.g., dysphagia or nutrition in prolonged coma. We included patients after they or their legal representative consented to participate. Patients were excluded if they had a known history of chronic gastrointestinal disease, bleeding, any kind of gastric surgery, lacked a stable circulation or had to be fasting for medical reasons on the day of measurement. We measured in the morning hours between 6 and 10 a.m. after the patient had been without enteral nutrition or water for at least 8 h. Then gastric content was aspirated from the nasogastric tube using a 50 ml syringe and BV was measured immediately afterwards. Subsequently, NF (Fresubin Energy Drink, Fresubin Original Fiber, Fresubin 2 kcal Drink, or Glucose 10%) was instilled via the feeding tube, mixed by alternating aspiration and injection until the mixture had a stable, homogeneous appearance. Then a small amount of the mixed fluid was aspirated again and the BV was measured (Figure 1). Gastric residual volume was then calculated according to the Equation (6). Besides the refractometric measurements, standard demographic data were evaluated and information on the use of opioids, catecholamines, sedatives, and prokinetics as well as data on the presence of pneumonia, the need for mechanical ventilation as well as the systemic immune response syndrome (SIRS) status was taken from the clinical records. The SIRS criteria were chosen because they are, in contrast to the sequential organ failure assessment (SOFA)—score, not biased by the level of consciousness in this special cohort on neurocritical care patients with acute primary brain disease.

### Calculation of GRV and Gastric Transport

The determination of calculated gastric residual volume (cGRV) and gastric transport from serial BV—measurements is essentially based upon the equations of Chang et al. (21, 25). Briefly, the BV of pure water is defined as zero. Physically, the BV correlates very well with the optical density of a solution. The BVs of single components in a solution are added accordingly to their proportion to the whole BV. Therefore, the BV of a mixture of water and NF can be calculated as follows:


(1) $$\begin{array}{l} BV\left(NF,\;H_{2}O\right)=a\times \;\frac{NF\;\left(ml\right)}{NF\;\left(ml\right)+H_{2}O\;\left(ml\right)} \end{array}$$

where “a” is a NF-product specific constant that can be accurately measured *in vitro* and that equals the BV of pure NF. The sum of the volumes of NF and H_2_O equals the GRV. Equation (1) describes the situation when NF is mixed with water in the absence of any other fluid. This situation can only occur *in vitro*, whereas *in vivo*, a certain amount of endogenously secreted “gastric juice” GF is mixed with the fluids added via the feeding tube.

To calculate the GRV and the unknown amount of NF it contains, BV is measured again after adding pure water to the GRV. If BV is measured at intervals that are sufficiently short, the gastric transport of solutes is negligible, whereas the GRV may be changed by adding water as desired. This leads to changes in volume concentration of solutes and, thus, changes in BV. Following these assumptions, Equation (1) can be rewritten as:


(2) $$\begin{array}{l} BV\times GRV\;\left(ml\right)=a\times NF\;\left(ml\right)=constant \end{array}$$

By adding water, the GRV increases, while the BV decreases. The product remains constant with BV_1_ and GRV_1_ representing values measured before, BV_2_ and GRV_2_ after adding water:


(3) $$\begin{array}{l} BV_{1}\times GRV_{1}=BV_{2}\times GRV_{2}\;=constant \end{array}$$

According to this definition, GRV_1_ is identical with the classical definition of GRV. To calculate the GRV, BV is measured before and directly after instilling and mixing a defined volume of water. For clarity, we will continue calculations assuming an added volume of 50 ml of water. It then can be stated that:


(4) $$\begin{array}{l} BV_{1}\times GRV=BV_{2}\times \left(GRV\;+50\;ml\right) \end{array}$$

Rearranging for GRV gives:


(5) $$\begin{array}{l} GRV=\frac{BV_{2}\;\times \;50\;ml}{BV_{1}-BV_{2}} \end{array}$$

For assessing the transport function of the stomach, it is necessary but not sufficient to measure GRV. In addition, the amount (or volume) of NF within the gastric content has to be measured as exact as possible at least once at a set time (e.g., 1 h) after instillation of a defined test bolus of NF. From Equation (1) it follows that the amount of NF can be calculated by


(6) $$\begin{array}{l} NF\;\left(ml\right)=\frac{BV_{1}\times GRV}{a} \end{array}$$

From the calculated differences in the volumes of pure NF present in the stomach, transport rates may easily be calculated by dividing with the time interval between time points of measurement.

## Results

### *In vitro* Formula Dilution Experiments: Refractometry

**Figure 2:**
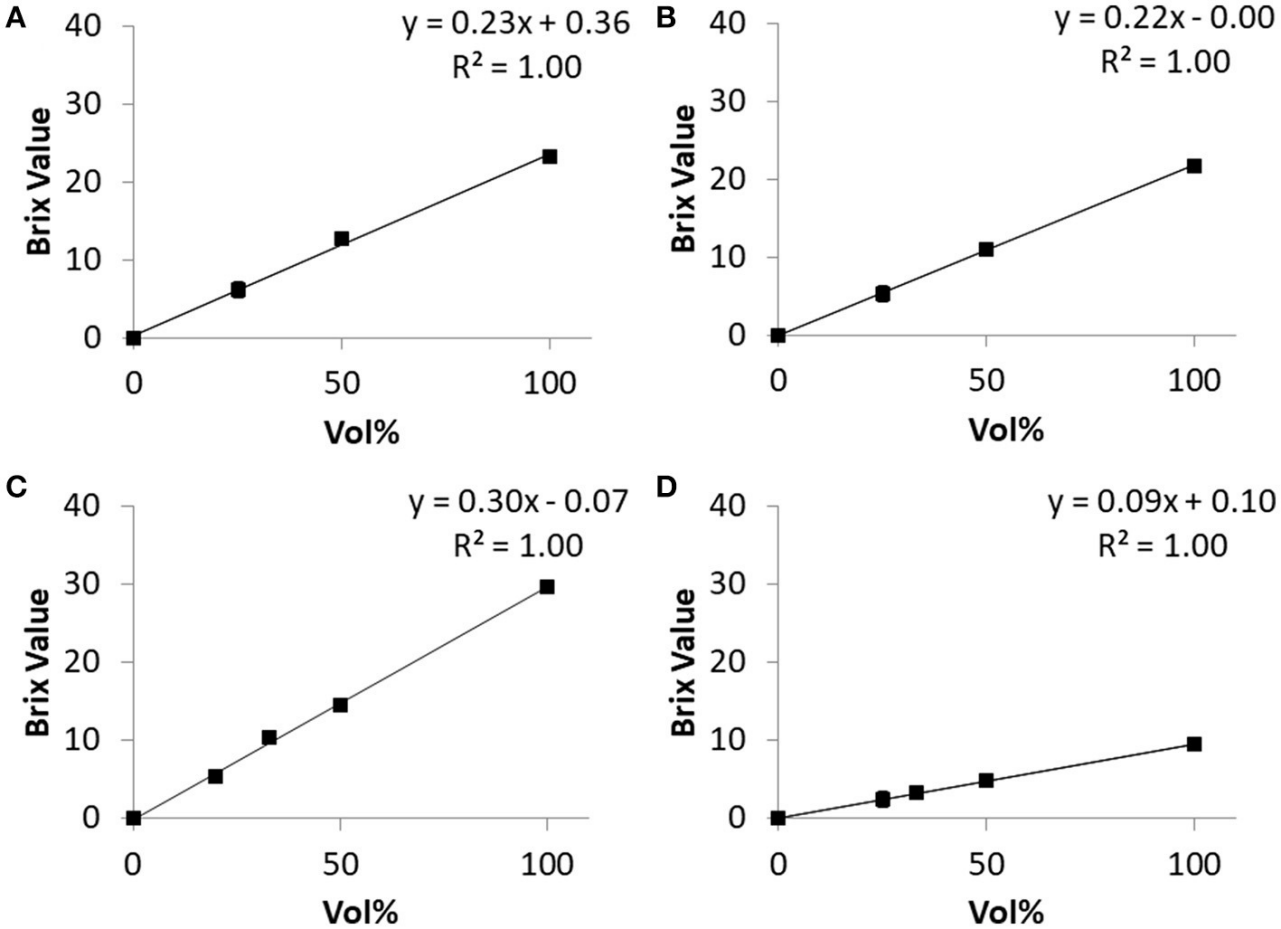
Relations of BV and content of four different solutions obtained *in vitro*. **(A)** Osmolyte HN, **(B)** Fresubin original fiber, **(C)** Diben Drink, **(D)** Glucose 10%. For each solution, three different persons performed the dilution and refractometric BV-measurement. Each dot represents the mean of these three measurements. The mean normalized standard deviation over all measured concentrations greater than zero was 3.4% for Osmolyte HN, 2.7% for Fresubin Original Fiber, 3.3% for Diben Drink, and 2.4% for Glucose 10%. The normalized standard error of the slope of the regression line was 3.67% for Osmolyte HN, 0.7% for Fresubin Original Fiber, 5.0% for Diben Drink, and 1.3% for Glucose 10%.

With the three commercially available NF-preparations tested in this study, a strict correlation between NF-concentration and BV was observed over the whole range of volume concentrations from zero to 100% in each single measurement of specific samples from single vials. The coefficient “a” of different commercial NF-products varied little between repeated experiments and raters (Figure 2).

**Figure 3:**
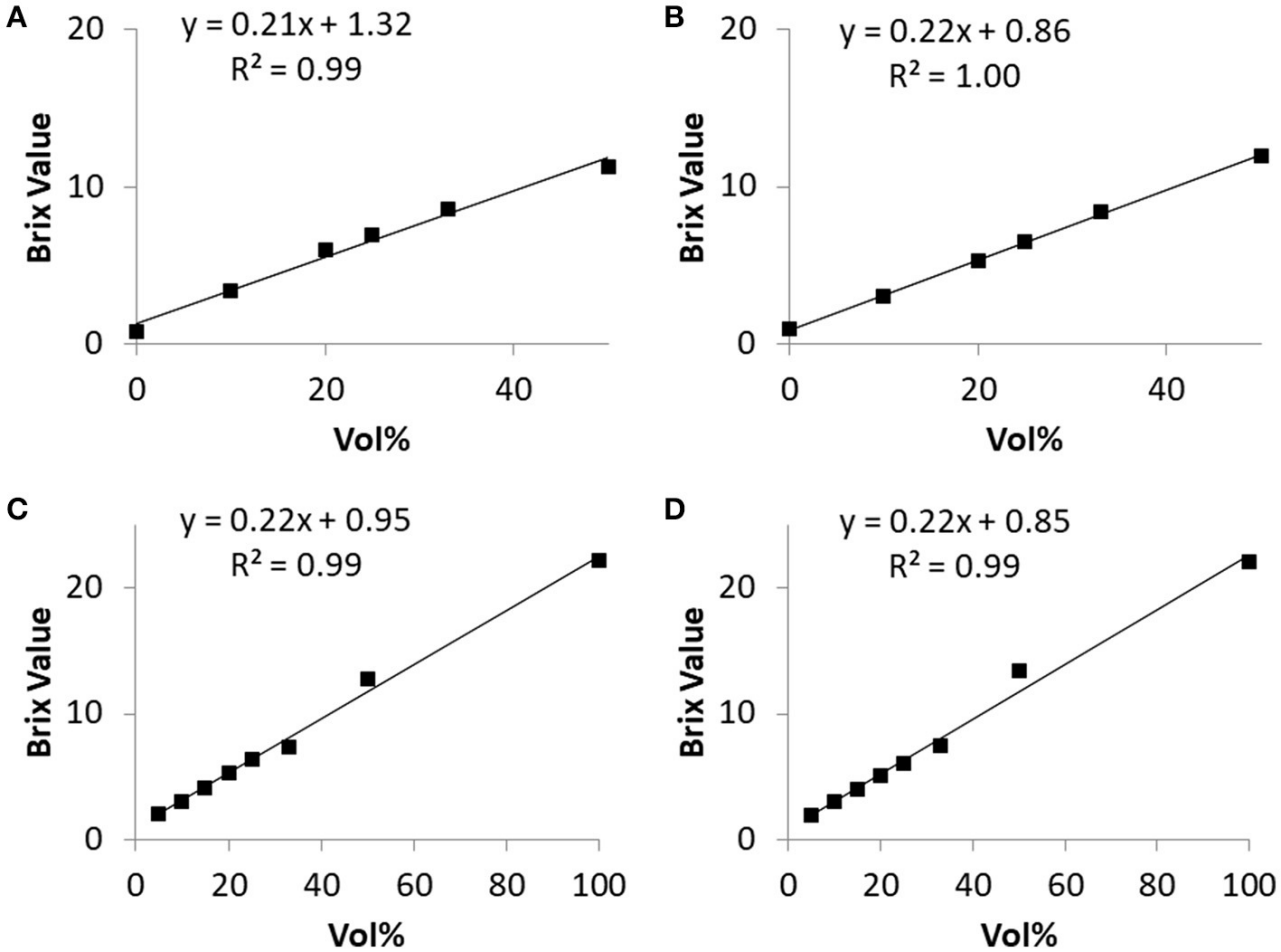
Relationship between Brix Value and nutrition formula concentration before and after 240-min incubation in simulated gastric fluid. **(A,B)** Fresubin Energy Drink before **(A)** and after **(B)** incubation. **(C,D)** Fresubin Original Fiber before **(C)** and after **(D)** incubation. Data points represent mean values of three **(A,B)** or two **(C,D)** independent samples, respectively. The normalized standard error of the slope of the regression line was 6.1% for **(A)**, 1.1% for **(B)**, 2.9% for **(C)**, and 3.9% for **(D)**.

To ensure that the precise estimation of BV is still possible under conditions mimicking *in vivo* conditions, the dependency of BV on NF concentration was repeated after 240 min of incubation of NF (“Fresubin Original Fiber” and “Diben Drink”) with SGF. The BV of SGF was 0.91 (*n* = 8, *SD* = 0.08). The strictly linear relationship was retained completely between 0 and 33% NF concentration and showed a moderate flattening at 50%, which was less pronounced after 240 min incubation than after 10 min (Figure 3). As expected from the high degree of correlation, the overall normalized standard deviation was small (e.g., 1.8% over 15 measurements with Diben Drink). For Fresubin Original Fiber, the normalized error of regression for defining the slope in Figures 2B, 3D after incubation with water or SGF was calculated as 0.5 and 3.9%, respectively.

### *In vitro* Formula Dilution Experiments: Glucose Concentration

**Figure 4:**
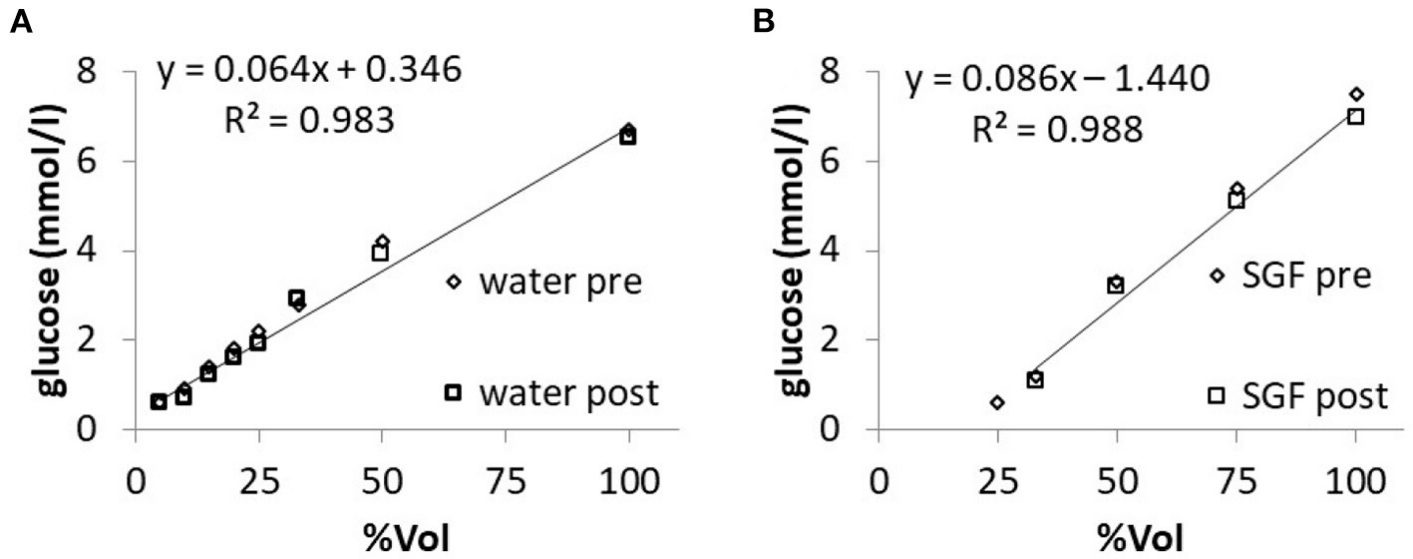
Relationship between measured glucose concentration and nutrition formula (Fresubin Original Fiber) concentration in water **(A)** or in SGF **(B)** before (“pre”) and after (“post”) 240-min of incubation in the respective medium; single measurements. Regression lines and equations are given for the “post” data. The normalized standard error of the slope of the regression line was 5.4% for **(A)** and 7.9% for **(B)** before incubation.

Molar glucose concentration was measured in “Fresubin Original Fiber” diluted with water to give the same volume concentrations of NF as tested in the refractometric measurements. The measured values of glucose concentration showed a linear correlation with the given volume concentrations. When SGF was used for dilution, the measured values of glucose concentration were lower compared to dilution with pure water (Figure 4). In mixtures with a proportion smaller than 33% NF, the handheld device gave a fault reporting. The same applied if mixtures with volume concentrations below 25% were tested after 240 min of incubation in SGF. Within the pure “Fresubin Original Fiber,” the device measured 6.62 mmol/l; SD: 0.5 mmol/l, *n* = 5, glucose concentration according to supplier: 7.8 mmol/l (personal communication). After dilution, the difference between reported and measured concentration decreased (for 33% volume concentration: calculated based upon datasheet: 2.6 mmol/l, measured value: 2.8 mmol/l). The normalized error of regression for the slope in Figures 4A,B after incubation with water or SGF was calculated as 7.9 and 6.1%, respectively.

### *In vitro* Formula Dilution Experiments: Protein Concentration

**Figure 5:**
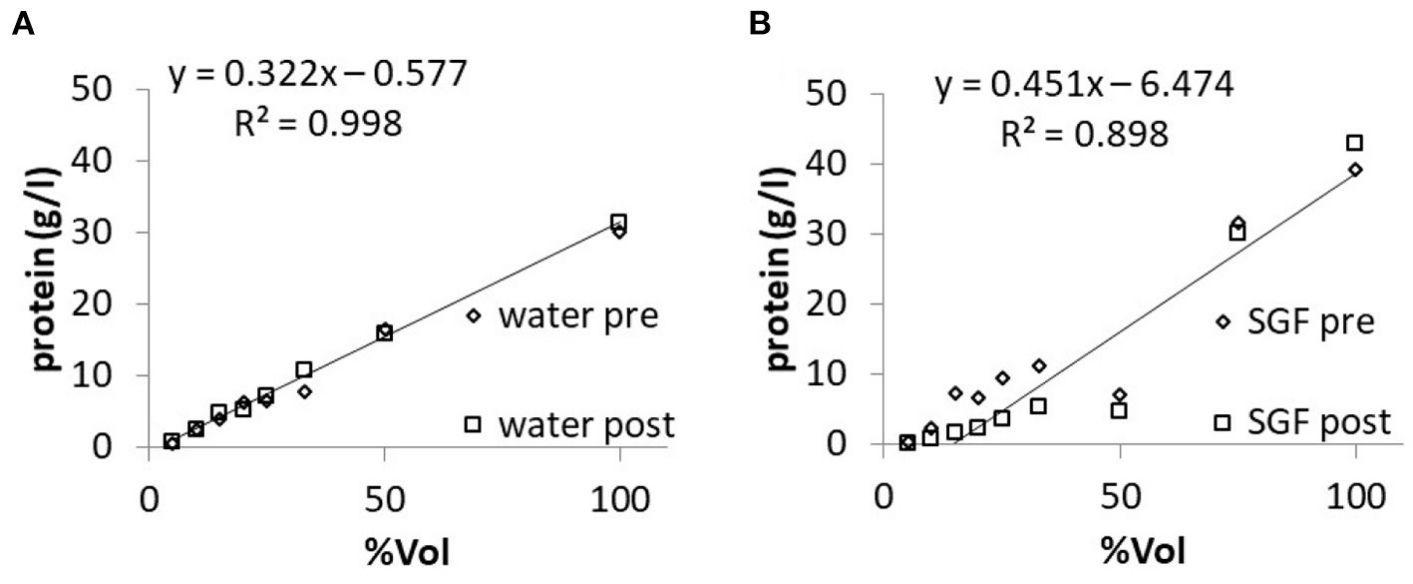
Relationship between measured protein concentration and nutrition formula (Fresubin original fibre) concentration in water **(A)** or in SGF **(B)** before (“pre”) and after (“post”) 240-min of incubation in the respective medium; single measurements. The normalized standard error of the slope of the regression line was 2.0% for **(A)** and 12.7% for **(B)** before incubation.

Dilution of NF (Fresubin original fibre) in water yielded linear reductions of the measured protein concentration. Dilution with SGF gave a non-linear increase with a prominent drop in the range of 50% volume concentration (Figure 5). With the same assay, the measured protein concentration was close to that reported by the manufacturer [32.6 g/l (SD: 0.4, *n* = 2) vs. 32 g/l] following dilution with water, whereas dilution with SGF resulted in reduced values of protein concentrations of only 9.95 g/l (SD: 1.6 mmol/l, *n* = 4). The respective measured concentrations in samples with ≥50% NF content did not change significantly over the time of incubation in either water or SGF. The normalized error of regression for the slope in Figures 5A,B after incubation with water or SGF was calculated as 7.8 and 15.7%, respectively.

### Cross Validation of Methods in Patient Samples

**Figure 6:**
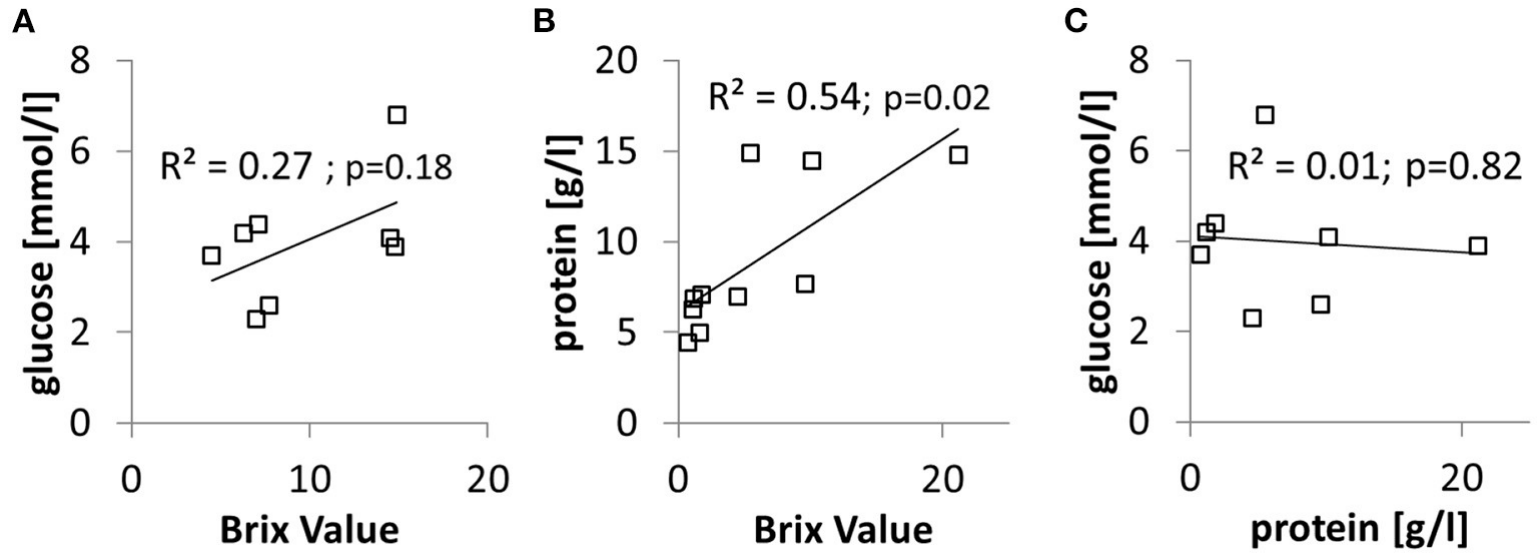
Sample of serially aspirated gastric juice were tested in parallel for BV, glucose, and protein concentration. **(A)** Relationship between glucose concentration and BV. **(B)** Relationship between protein concentration and BV. **(C)** Relationship between glucose concentration and protein concentration.

We obtained samples of aspirated gastric content from two patients which were included in the study protocol described in the following section. For protein and glucose testing, aliquots (2 ml) from these samples were separated from the material that was immediately analyzed via bedside refractometry. Thereby we could test these samples in parallel with the three methods described in the previous sections on *in vitro* experiments. The results are shown in Figure 6. While Refractometry and Bradford tests for protein concentrations gave defined values for all of the eight samples, glucose testing using the point-of-care device failed in two samples. The BV correlated weakly with measured glucose and significantly with protein concentration in samples. In contrast, there was no correlation between glucose and protein concentrations in samples. Inevitably, the same applied for of the calculated GRV and NF-content in samples (data not shown).

### Refractometric Measurements of GRV and Gastric Transport in Patients

Since superiority of refractometric estimates of gastric volumes and contents was demonstrated in the *in vitro* experiments, glucose and protein measurements were abandoned and only refractometric measurements were evaluated further on. Serial measurements of GRV and NF-concentration were performed in patients submitted to the neurological intensive care unit of the Rostock University Medical Center. From all 28 patients meeting the aforementioned inclusion- and exclusion-criteria, basic clinical and demographic parameters were recorded. Table 1 summarizes essential information on the studied patient sample. First, we categorized ischemic stroke regarding the major affected territory. Then we reviewed the radiologic images and categorized right/left and bilateral stroke, regardless of supra- or infra-tentorial localization or ischemic vs. hemorrhagic genesis. The median number of days between admission and refractometric measurements was 6 days (IQR 4–14). Overall, six patients (24%) fulfilled at least one criterion of our definition of feeding intolerance, namely nausea, vomiting, reflux of gastric contents via the feeding tube, or treatment with prokinetics (e.g., erythromycin or domperidone).

**Table 1:** Demographic characteristics and clinical parameters at the time of measurement.

	**No. of patients; % of sample or mean ±SD**
Total sample	28 (100%)
Female	11 (39%)
Age	73 ± 11 years
Stroke	24 (86%)
Cerebral ischemia	12 (43%)
Brain stem ischemia	5 (18%)
Cerebral hemorrhage	5 (18%)
Brain stem hemorrhage	2 (7%)
Left	11 (39%)
Right	9 (32%)
Bilateral	4 (14%)
NIHSS at admission	17.7 ± 8.7
Status epilepticus	3 (11%)
Acute inflammatory polyneuropathy	1 (4%)
Pneumonia	21 (75%)
SIRS	17 (61%)
Diabetes mellitus	14 (50%)
Polyneuropathy	6 (21%)
On mechanical ventilation	13 (46%)
Receiving noradrenaline	7 (25%)
Receiving opioids	15 (54%)

*NIHSS, National Institute of Health Stroke Scale*.

Refractometric GRV measurements were started after an 8-h period of fasting. In a first step, it was tried to draw an initial aspirate from the feeding tube. In eight patients, this was possible (3 × 4, 1 × 6, 1 × 7, 1 × 10, 1 × 36, 1 × 50 ml), altogether giving a median fasting residual volume of 7. Then, if possible, baseline GRV was estimated by two BV-measurements within <5 min, the first after application of 50 ml NF, followed by thorough mixing. After adding and mixing additional 50 ml of water, the second BV was measured. The median calculated GRV was 62 ml (IQR: 45–104 ml). By applying the experimentally estimated constant “*a*” according to the aforementioned Equations (1) and (6), it was then possible to calculate the median gastric NF content which was lower than the volume of instilled NF (median: 32 ml; IQR: 26–44 ml).

One hour later, a second GRV was calculated by another short-interval measurement of two BV, one before and one after application of water. In 11 patients, it was then not possible to draw a sample, so additional volumes of NF and water were applied and later on subtracted from the calculated GRV and NF content values. With these corrections, median calculated GRV was 25 ml; (IQR: −6–51 ml). The remaining gastric NF content was calculated to be 8 ml (IQR: −13–17 ml). There was a significant decline of the calculated GRV and NF content within the 1-h interval (Wilcoxon-Rank-Test, *p* < 0.001 for both GRV and NF content). From this data, a median volume transport rate of 93 ml/h (IQR: 56–105 ml/h) and a median rate of nutrition transport of 34 ml/h (IQR: 15–42 ml/h) were calculated. When considering the differences in energy content of the used commercial NF products, the calculated rate of transported calories was 18 kcal/h (IQR: 11–39 kcal/h).

### Associations Between Gastric Transport and Clinical Parameters

Differences between sexes in transport rates could not be seen, but higher age was correlated with increased rates of energy transport (Rho 0.61, *p* < 0.001).

We could not detect a difference in transport rates between patients with or without stroke, but energy transport significantly differed between bilateral stroke, right- and left-sided stroke (Kruskal-Wallis-test, *p* = 0.016). Also right-sided lesions were associated with lower energy transport rates than left-sided (Mann-Whitney-test, *p* = 0.02). There was no significant difference between ischemic and hemorrhagic or between hemispheric and brain stem strokes. Although not statistically valid due to the small subgroup size, patients treated with prokinetics (4/28) had gastric transport rates even lower than patients w/o prokinetics (median volume transport: 27 vs. 95 ml/h; median food transport: 5 vs. 37 ml/h).

Whereas, the presence of SIRS-criteria or a diagnosis of diabetes mellitus were not significantly related to altered nutrition transport, increased blood glucose levels were inversely correlated with energy transport (Rho: −0.391; *p* = 0.039). The same applied to patients on mechanical ventilation vs. spontaneously breathing patients (median 12 vs. 26 kcal/h, *p* = 0.017). In patients receiving sufentanil, a significant dose-dependent inverse correlation with energy transport rates was observed (Rho −0.514; *p* = 0.005).

### Prediction of Feeding Intolerance

**Figure 7:**
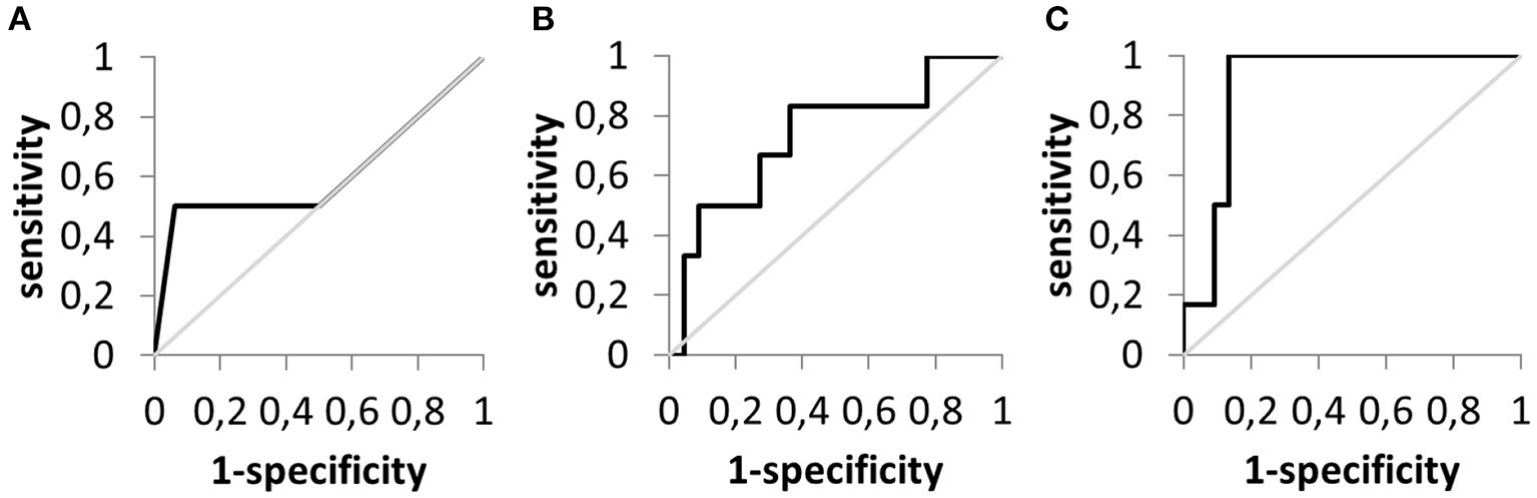
Receiver operating characteristic curves to evaluate the prediction of feeding intolerance. **(A)** Aspirated gastric residual volume (*n* = 20). **(B)** Calculated gastric residual volume (*n* = 28). **(C)** Calculated nutrition formula transport (*n* = 28).

To analyze the value of the measured volume and transport parameters for predicting signs of feeding intolerance, receiver operating characteristic (ROC) curves were plotted (Figure 7).

Table 2 summarizes the respective findings. Whereas, the mere testing for residual volume by aspiration in patients after fasting had the lowest predictive power, parameters derived from refractometry and especially measurements of food transport are clearly eligible to identify patients with feeding intolerance. This finding is underlined by the results of the ROC-analysis. A food transport rate of more than 20 ml/h virtually excluded the presence of feeding intolerance in the studied patient sample.

**Table 2:** Evaluation of parameters of gastric emptying as classifiers for feeding intolerance.

	**AUC**	***p*-value**	**Cut-off**	**sensitivity**	**specificity**
aGRV↑	0.61	0.51	35 ml	0.5	0.94
cGRV↑	0.74	0.08	69 ml	0.83	0.67
Volume transport↓	0.76	0.06	50 ml/h	0.67	0.91
Food transport↓	0.90	0.003	20 ml/h	1	0.86
Energy transport↓	0.76	0.06	12 kcal/h	0.67	0.86

*Results of receiver operating characteristic curves: AUC, area under the curve; aGRV, aspired GRV; cGRV, calculated GRV; volume transport, change in volume as calculated from serial refractometry; food transport, change in NF volume in cGRV; energy transport, change in the energy content within cGRV. Up/down arrows depict increases or decreases of the respective parameters*.

## Discussion

Despite the well-recognized importance of altered gastrointestinal motility in critically ill patients, there is an ongoing need for easily applicable and valid methods to assess it. Gastric residual volume estimated by simple aspiration has been shown to be of limited clinical significance, whereas more precise and elaborate measures of gastric function (e.g., scintigraphic or breath tests) have not yet been implemented widely as routine procedures in critical care medicine. With the current work, we reproduce previous single-center works that suggested refractometry as an option for monitoring enteral feeding which is more precise than the still widely used aspiration-based GRV and far still less demanding than the abovementioned methods. Furthermore, from the obtained data we conclude that it has the potential to enhance the study of gastric dysmotility as a consequence of autonomic disorders in stroke patients beyond merely nutritional aspects.

Compared to other dilution assays which might be implemented in point-of-care settings, refractometry offers superior precision and stability. Due to the clinical characteristics of the patients included, a high prevalence of gastrointestinal dysmotility could be expected. To name just a few, increased blood glucose levels, general disease severity, and the use of opioids and sedatives have been proposed to impair gastric function (33–38). As a proof-of-concept, refractometry-based measurements could reproduce the known interference of mechanical ventilation, opioids, and blood glucose levels with gastric transport.

Especially in patients with underlying neurological diseases, critical illness, or impaired consciousness of any cause, monitoring of transport is crucial for reducing the risk of aspiration by preventing gastric overload in the course of clinical nutrition. Furthermore, it allows a safe and fast replacement of intravenous alimentation by enteral feeding in patients with stable motility.

Despite the proven reliability of refractometry, some results of our study require critical reflection and clarification.

First, physically implausible negative cGRV were calculated in seven patients at the time of the second measurement, after subtracting the volume of added H_2_O and NF. Thorough mixing by gently alternating aspiration and extrusion via the syringe and the feeding tube, meanwhile taking care not to adhere at the gastric mucosa during aspiration, may take a couple of minutes. Parts of the added volume might already have passed the pylorus before the refractometric measurements were completed. In our opinion, this is the most likely explanation for negative values of the corrected GRV. From a practical perspective, this would indicate a rather vigorous gastric motility and would virtually exclude feeding intolerance due to upper gastrointestinal dysfunction. This hypothesis is supported by the fact that such negative corrected GRV only occurred in patients from whose feeding tubes no fluid could be aspirated before the second measurement period and for whom thus additional applications of water and NF were required to measure the BV.

In line with the hypothesis of gastric transport occurring during the in-between measurement is the fact that median estimated first GRV was also lower than the cumulative added fluid volume. This finding has already been reported in the early validation studies for refractometric GRV—measurements (26).

Second, in two patients, a negative, i.e., reverse transport of NF-volume was calculated. Retrograde transfer of NF or another medium with a sufficient refractive index from the duodenum might explain these results. This phenomenon has indeed been described in the literature already—even in healthy subjects—and is known to accompany upper gastrointestinal dysmotiliy and vomiting (11, 39–41).

**Figure 8:**
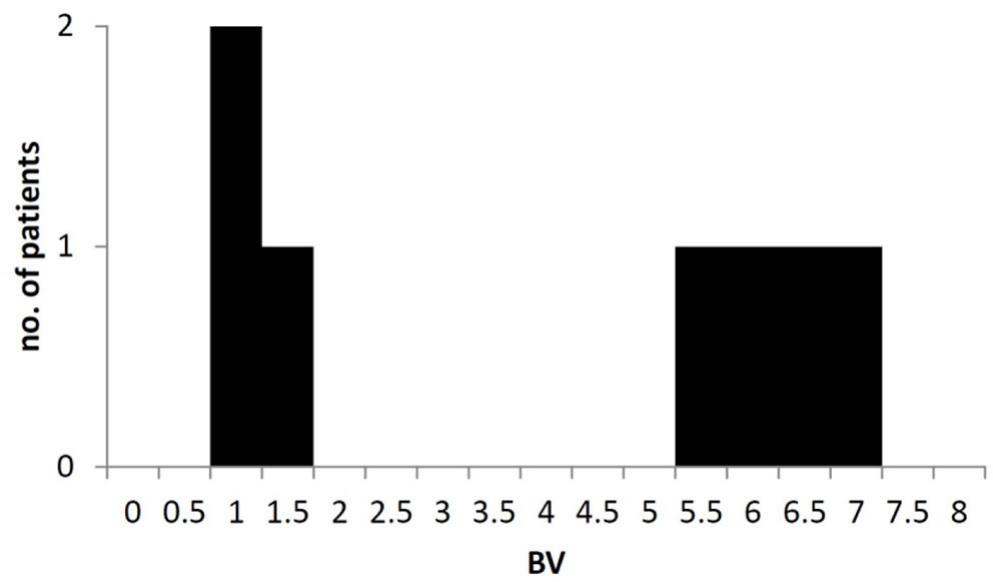
Bimodal distribution of Brix Value in aspirates of gastric content after fasting. Brix values >3 most likely reflecting the presence of remnants from nutrition formula or other ingested solutes within the stomach in some patients.

Third, it may be argued that the BV of endogenously produced gastric juice may add a relevant bias to the calculations. Seemingly, the broad range of BV estimated in initial aspirates may support this conclusion. However, it is more likely that even after the 8-h fasting period, some NF content is still present in the stomach. Median gastric half-emptying times between 2 and 3 h in critically ill patients are not uncommon and have been reported previously in patient samples studied with scintigraphy and breath tests (42, 43). So it is plausible that between 6% (assuming 4 × *T*_0.5_ = 120 min) and 16% (assuming 3 × *T*_0.5_ = 180 min) of the gastric contents taken up before the beginning of the fasting period might still remain in the stomach at the time of the second measurement. The bimodal distribution of fasting BV (Figure 8) would thus reflect the presence or absence of NF-remnants in gastric content aspirates with BV between 5 and 7.4 (*n* = 5), whereas the sample values of the other three patients are within the range of values we have been measured for SGF and those reported for pure fasting gastric juice in the literature (21, 23). This consideration implies a practical consequence: irrespective of the BV found in initial aspirates, subsequent serial BV measurements and calculations of NF-content and transport will still give proper results if the same NF-product is used for testing as has been used for feeding previously.

Although refractometry cannot be used to distinguish endogenous gastric or intestinal secretions (in contrast to glucose and protein measurements), we could prove its robustness during *in vitro* experiments. We could confirm its clinical significance as known risks for gastric dysfunction (high blood glucose, opiods, mechanical ventilation) were associated with lower calculated transport rates. In addition, differences associated with stroke sidedness were seen, although these results have to be interpreted with great caution due to the low patient number. The same applies to the observed reduction in transport rates in the four patients on prokinetics which may not be overinterpreted but may simply reflect a reaction of the medical ICU staff to subtle clinical signs of gastric dysmotility. The fact that prokinetics evidently did not effectively restore gastric transport is in line with previous reports on their unsatisfactory efficiency in real world settings (44, 45).

Taken together, The advantage of low costs and the possibility to measure GRV and food transport whenever desired and independently from technical requirements that are often not met in stroke medicine and neurointensive care make refractometry a promising tool to study the yet ill-defined relations between brain lesions, autonomic dysfunction, and altered motility in stroke patients (46–48).

## Data Availability Statement

The raw data supporting the conclusions of this article will be made available by the authors, without undue reservation.

## Ethics Statement

The studies involving human participants were reviewed and approved by Ethics Committee of the Medical Faculty University of Rostock. The patients/participants or their legal representatives provided their written informed consent to participate in this study.

## Author Contributions

RP conceptualized the study. RP, MK, and SK recruited participants. MK and RP performed the measurements, data collection and analysis. TS and KP performed additional *in-vitro* measurements. RP, MW, and MK prepared the manuscript. All authors approved the final version.

## Conflict of Interest

The authors declare that the research was conducted in the absence of any commercial or financial relationships that could be construed as a potential conflict of interest.

## Publisher's Note

All claims expressed in this article are solely those of the authors and do not necessarily represent those of their affiliated organizations, or those of the publisher, the editors and the reviewers. Any product that may be evaluated in this article, or claim that may be made by its manufacturer, is not guaranteed or endorsed by the publisher.

## Supplementary Material

Supplementary Figure 1 Theoretical overestimation of NF-volume in mixtures of 50 ml NF with 50 ml of water and a variable amount of endogenous gastric secrete (“gastric juice”) with a variable refractive index (BV). The presence of gastric secretions with a BV higher than 0 leads to an overestimation of the volume of NF present within the investigated gastric volume. The amount of overestimation is shown as a function of the BV of gastric secretions and of the amount of gastric juice present within the stomach for a standard measurement which is done by applying 50 ml of water to a GRV in which 50 ml of NF are truly present.

## Abbreviations

BV: Brix value (refractive index)
cGRV: calculated gastric residual volume
GRV: gastric residual volume
HCl: hydrochloric acid
IQR: interquartile range
NF: nutrition formula
ROC: receiver operating characteristic
SGF: simulated gastric fluid
SIRS: systemic inflammatory response syndrome
SOFA: sequential organ failure assessment score.
