# Supplementary figures and images for: Serial Measurements of Refractive Index, Glucose and Protein to Assess Gastric Liquid Nutrient Transport—A Proof-of-Principal Study
Source: Front Nutr. 2022 Feb 3;8:742656. doi: 10.3389/fnut.2021.742656 (PMC8850719; doi:10.3389/fnut.2021.742656)

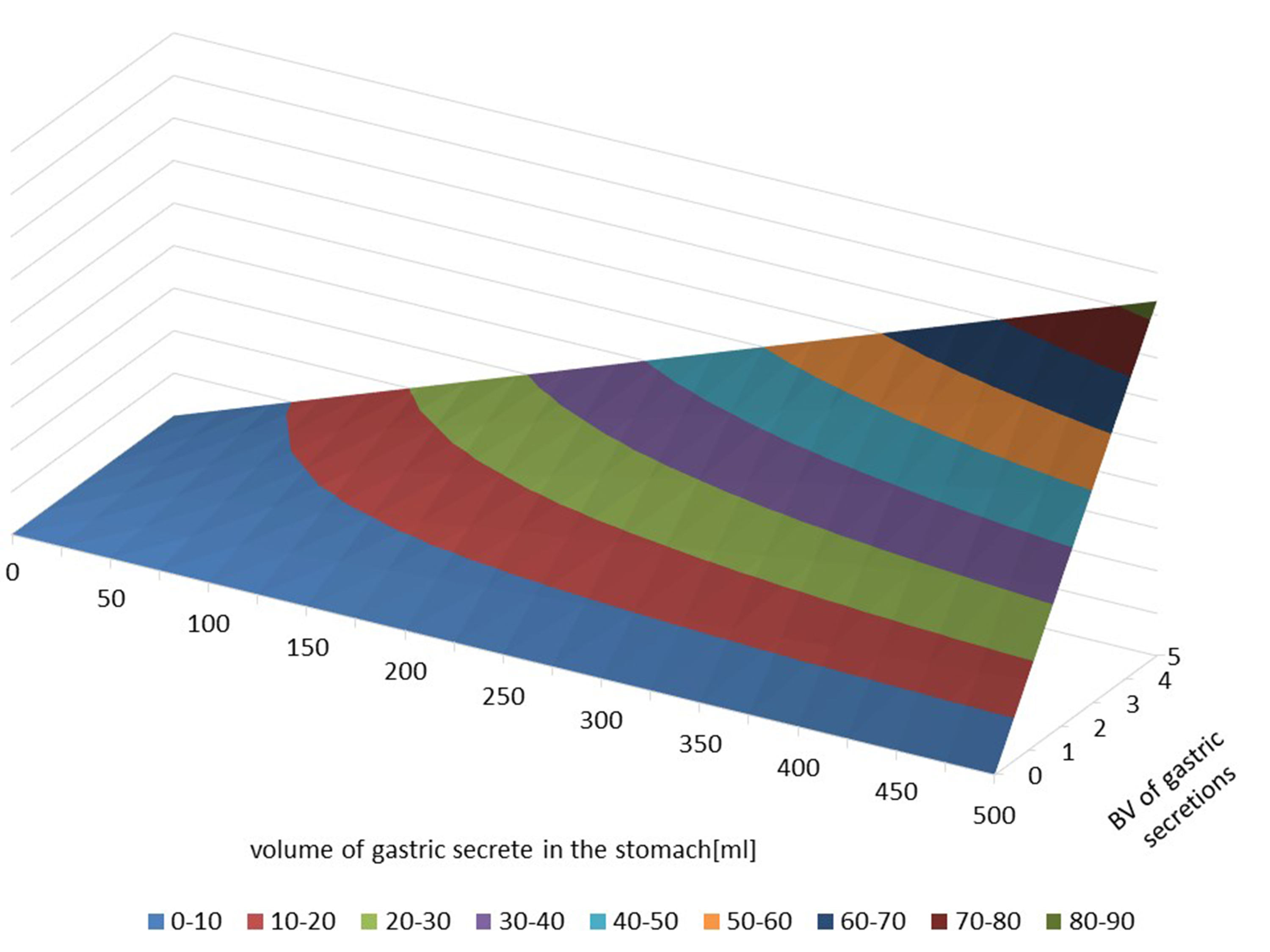

Supplement: Supplementary Figure 1 — Theoretical overestimation of NF-volume in mixtures of 50 ml NF with 50 ml of water and a variable amount of endogenous gastric secrete (“gastric juice”) with a variable refractive index (BV). The presence of gastric secretions with a BV higher than 0 leads to an overestimation of the volume of NF present within the investigated gastric volume. The amount of overestimation is shown as a function of the BV of gastric secretions and of the amount of gastric juice present within the stomach for a standard measurement which is done by applying 50 ml of water to a GRV in which 50 ml of NF are truly present. [file Image_1.JPEG]
